# Effectiveness of Different Acceleration Methods for the En Masse Retraction of Upper Anterior Teeth: A Systematic Review and Meta-Analysis

**DOI:** 10.7759/cureus.105562

**Published:** 2026-03-20

**Authors:** Mudar Mohammad Mousa, Mohammad Y Hajeer, Mohamad J Othman, Mohammad Khursheed Alam, Ahmad Salim Zakaria, Alaa Oudah Ali Almusawi

**Affiliations:** 1 Department of Orthodontics, Faculty of Dentistry, Damascus University, Damascus, SYR; 2 Department of Preventive Dentistry, College of Dentistry, Jouf University, Sakakah, SAU; 3 Department of Orthodontics, School of Dental Sciences, Universiti Sains Malaysia, Kota Bharu, MYS; 4 Department of Orthodontics, Faculty of Dentistry, Al-Kunooz University, Basrah, IRQ

**Keywords:** corticotomy, en masse retraction, low‑level laser therapy, orthodontic tooth movement, systematic review, treatment acceleration

## Abstract

En masse retraction of the anterior teeth following first premolar extraction is a common orthodontic approach for correcting Class II malocclusion and bimaxillary protrusion. Several acceleration techniques have been proposed to reduce treatment duration, yet their comparative effectiveness remains uncertain. This systematic review evaluated randomized controlled trials published between January 1995 and May 2025 that investigated surgical, physical, mechanical, biochemical, hormonal, and pharmacological methods for accelerating en masse retraction in healthy orthodontic patients, with outcomes reported as the rate of retraction in millimetres per month. Six electronic databases and manual reference searches were screened, and the risk of bias was assessed using the Cochrane risk of bias 2 (RoB 2) tool. Nine trials involving 347 participants (mean age 21.6±2.97 years) met the inclusion criteria. One study was rated as high risk of bias, four had some concerns, and the remainder were rated as low risk. Flapless corticotomy increased the retraction rate by an average of 0.24 mm/month compared with conventional treatment (moderate‑quality evidence), while its combination with low‑level laser therapy produced the greatest and most sustained acceleration, achieving a 43.8% higher rate than conventional protocols (high‑quality evidence). Corticoalveolar perforations increased the rate by 0.28 mm/month (low‑quality evidence), and low‑intensity electrical stimulation accelerated retraction by approximately 28% (low‑quality evidence). Platelet‑rich plasma injections showed no meaningful benefit. Overall, flapless corticotomy, particularly when combined with low‑level laser therapy, appears to be a promising method for accelerating en masse retraction, offering clinically relevant reductions in treatment duration. However, these findings should be interpreted with caution due to the limited and heterogeneous evidence base, with most techniques supported by low‑to‑moderate quality evidence. The current data do not allow definitive conclusions regarding the superiority of one technique over another.

## Introduction and background

The extraction of first premolars is one of the most common procedures for camouflaging Class II cases and treating bimaxillary protrusion cases, where the extraction space can be closed by either en masse or two-step retraction of the six anterior teeth [1]. En masse retraction refers to the simultaneous distal movement of the six anterior teeth (canine to canine) as a single unit to close the extraction spaces, as opposed to the two-step method, where canines are retracted first, followed by the incisors [1-3]. This technique reduces the force on the anchorage unit and therefore requires simple procedures to achieve adequate anchorage [4]. On the other hand, the en masse retraction technique is considered more beneficial for shortening treatment time after premolar extraction, but it requires more complex anchorage [1,5].

Despite the numerous benefits of orthodontic treatment, the duration of treatment is often a primary reason patients hesitate to proceed or decline treatment [6]. The length of orthodontic treatment is a significant concern, with fixed appliances typically requiring two to three years, including follow-up and retention phases [7]. Consequently, while many patients prefer a shorter treatment duration of 6-12 months, expectations vary based on individual factors such as age, malocclusion severity, and personal circumstances [8]. The prolonged duration of treatment may also predispose patients to complications, including pain, discomfort, root resorption, periodontal disease, and dental caries [9-12].

Acceleration methods in orthodontics have undergone significant advancements, particularly through surgical techniques such as traditional corticotomy and piezo-assisted corticotomy, which aim to enhance tooth movement by inducing a localized inflammatory response known as the regional acceleratory phenomenon (RAP), which temporarily increases bone remodeling, both of which have demonstrated promising outcomes [13,14]. Other approaches include laser-assisted corticotomy and corticision [15-17]. Additionally, physical methods were examined, including low-energy lasers [18], pulsed electromagnetic fields, and low-intensity direct current, which may influence cellular activity in the periodontal ligament [19,20]. Hormonal and pharmacological approaches, including platelet-rich plasma, have also attracted attention [21].

Biomechanical strategies to reduce friction between orthodontic wires and brackets are critical for accelerating dental movement [22]. Over the years, multiple generations of metal brackets have been developed, culminating in the introduction of self-ligating brackets [23,24]. Moreover, segmenting the dental arch into anterior and posterior sections can help reduce friction and minimize force loss, thereby accelerating orthodontic treatment [25].

Tunçer et al. conducted a randomized controlled trial (RCT) to compare the en masse retraction of upper anterior teeth using flapless corticotomy with conventional en masse retraction. They reported that the average retraction time was 9.33±4.10 months in the flapless corticotomy group, compared with 9.27±2.55 months in the conventional retraction group. Their findings showed no statistically significant differences between the two groups' rates [26]. In contrast, Hatrom et al. also compared the en masse retraction of upper anterior teeth using flapless corticotomy with conventional en masse retraction. They found that the flapless corticotomy group had a significantly greater retraction rate of 1.2 mm/month compared with 0.6 mm/month in the control group [27]. Additionally, an RCT investigated the effect of low-level laser therapy (LLLT) in combination with self-ligating brackets and conventional brackets, compared with conventional treatment during en masse retraction [28]. They reported that the tooth movement rate was 0.635 mm/month in the LLLT group using self-ligating brackets, 0.6625 mm/month in the LLLT group using conventional brackets, and 0.4875 mm/month in the control group. This demonstrates that the tooth movement rate was significantly greater in the LLLT group than in the control group, although no statistically significant difference was observed between the bracket types used. Therefore, the results of the different studies are inconsistent, and no valid conclusions can be drawn from the current trials regarding en masse retraction when using the acceleration of orthodontic treatment.

A literature review revealed that no systematic review has compared various acceleration methods for the en masse retraction of the six anterior teeth; instead, existing reviews have primarily focused on canine retraction alone. Therefore, it is essential to conduct this systematic review to determine the most effective method for accelerating the en masse retraction of the upper anterior teeth in cases involving camouflaging malocclusion associated with the extraction of the first premolars. The primary focused review question was: "What is the available evidence regarding the effectiveness of different methods for accelerating en masse retraction during orthodontic treatment to camouflage malocclusions that necessitate the extraction of first premolars?" This review is registered in PROSPERO (#CRD420251127529).

## Review

Materials and methods

Scoping Search

Before initiating this review, a preliminary PubMed search was conducted to verify that no existing reviews on the same topic were available. The search results confirmed the absence of similar reviews on the en masse retraction of anterior teeth in malocclusion patients requiring the extraction of first premolars. In addition, the preliminary search indicated that sufficient clinical trials were available for inclusion in this analysis. This systematic review followed the Preferred Reporting Items for Systematic Reviews and Meta-Analyses (PRISMA) guidelines [29].

Search Strategy

An electronic search was performed to identify articles related to the accelerated en masse retraction of maxillary anterior teeth from January 1995 to May 2025. The electronic search was conducted using PubMed, Web of Science, Scopus, the Cochrane Central Register of Controlled Trials, Embase, and Google Scholar. Due to the broad and unstructured nature of Google Scholar, the first 200 results (sorted by relevance) were screened, and additional relevant articles were identified by manually reviewing the reference lists of included studies to minimize selection bias. The keywords used in the search strategy are listed in Table 1. Details of the electronic search strategy are provided in appendix 1.

**Table 1:** Keywords used in the electronic search. RAP: regional acceleratory phenomenon; PAOO: periodontally accelerated osteogenic orthodontics; LLLT: low-level laser therapy; HELT: high-energy laser therapy; YAG: yttrium-aluminum garnets; YSGG: yttrium scandium gallium garnet

Aspect of the search query	Keywords
Orthodontics	Orthodontic, tooth movement, orthodontic tooth movement, tooth displacement, orthodontic treatment, orthodontic therapy.
Retraction of anterior teeth or space closure	En masse retraction, extraction therapy, front retraction, orthodontic gap closure, orthodontic space closure.
Acceleration method	Fast, rapid, duration, velocity, speed, short, RAP, acceleration, periodontally accelerated, orthodontic osteogenesis, PAOO, minimally invasive, piezosurgery, piezo, piezoelectric, piezocision, piezotome, piezopuncture, lasercision, corticopuncture, microsurgery, micro-incisions, micro osteoperforations, micro perforations, perforations, corticision, laser, high intensity laser therapy, HELT, hard laser, high-energy laser, Erbium lasers, Er: YAG laser, Er, Cr: YSGG laser, Nd: YAG laser, laser-assisted corticotomy, laser-assisted, laser induced, laser decortication, low-level laser therapy, low-intensity laser, LLLT.

First, two review authors (MMM and MJO) assessed the articles' titles and abstracts to determine whether they met the entry criteria. Then, the full text of all articles that met the criteria was assessed to see if they could be included in the review, and any article that did not meet one or more of the inclusion criteria was excluded. Any conflict between the reviewers was resolved by a third reviewer (MKA).

Inclusion and Exclusion Criteria

The inclusion criteria were defined using the Participants, Intervention, Comparison, Outcomes, and Study Design (PICOS) framework. Participants included healthy patients of both sexes, aged 16-35 years, without restrictions on race, who required first premolar extractions for en masse retraction, had complete dentition except for third molars, and had not received prior orthodontic treatment. Intervention was en masse retraction of the six anterior teeth assisted by surgical, physical, mechanical, biochemical, hormonal, or pharmacological acceleration methods. Comparison involved en masse retraction of the anterior teeth using an alternative acceleration method or without any acceleration. Outcomes included the rate of tooth movement (RTM) and overall treatment duration as the primary measures. Study design was limited to randomized controlled trials (RCTs) published between January 1995 and May 2025. The inclusion and exclusion criteria for study selection are elaborated in Table 2. Retrospective and laboratory studies, non-randomized trials, single clinical cases, split-mouth clinical studies, survey studies, personal opinions, or studies with fewer than 10 patients were excluded.

**Table 2:** Inclusion and exclusion criteria according to the PICOS design framework. PICOS: Population, Intervention, Comparator, Outcomes, and Study; N/A: not applicable

PICOS framework	Inclusion criteria	Exclusion criteria
Population	Healthy patients aged 16-35 years requiring first premolar extractions for en masse retraction were included.	Patients younger than 16 years of age or older than 35 years. Patients with previous orthodontic treatment.
Intervention	Studies investigating surgical, physical, mechanical, biochemical, hormonal, or pharmacological acceleration methods during en masse retraction were included.	Studies investigating acceleration methods for canine retraction only. Studies focusing solely on the alignment phase.
Comparison	Studies comparing an acceleration method to another acceleration method or to a non-accelerated treatment were included.	N/A
Outcomes	Studies reporting tooth movement rate (mm/month) or treatment duration as primary outcomes were included.	Studies not reporting quantitative data on retraction rate.
Study design	Randomized controlled trials, published between January 1995 and May 2025.	Non-randomized trials. Cohort studies, case-control studies, cross-sectional studies, case reports, case series reports, editorials, commentaries, and technical notes.

Data Collection Process

Two reviewers (MMM and MYH) collected data from the selected studies and organized it into tables. In instances of disagreement, the third author (MKA) intervened to facilitate a consensus. The tables include key details such as general information (author names, study setting, and publication year), methodologies (study design), participant demographics (sample size, age, and malocclusion type), intervention characteristics (type of acceleration method, appliance specifications, and force magnitude), follow-up duration, and primary outcomes (rate of tooth movement and treatment duration).

Evaluation of the Risk of Bias

The risk of bias was assessed using the RoB2 tool for the included randomized controlled trials (RCTs) [30]. The evaluation employed the terms "low risk," "high risk," and "some concerns" to categorize bias across various domains: bias arising from the randomization process, bias due to changes in the planned intervention, bias due to incomplete outcome data, bias in outcome measurement, and bias in the selection of reported results. The overall judgment regarding the risk of bias in the selected studies was determined as follows: a "low risk of bias" designation was given when all domains were rated as "at low risk of bias;" "some concerns" were noted if at least one domain was categorized as "some concerns" without any domain rated as "high risk of bias;" and "high risk of bias" was indicated if one or more domains were rated as "at high risk of bias" or if multiple domains showed concerns that significantly undermined confidence in the results.

Data Synthesis

The meta-analysis was performed using RevMan software, version 5.4 (Copenhagen, Denmark: Nordic Cochrane Center, Cochrane Collaboration). For continuous outcomes, a random-effects model with inverse-variance weighting was used, with results expressed as mean differences and 95% confidence intervals (CIs). Statistical heterogeneity was assessed using the I² statistic, interpreted according to Cochrane guidelines (30-60%: moderate, 50-90%: substantial, 75-100%: considerable). Heterogeneity was evaluated using I² values rather than the chi² p-value alone, given the limited power of the latter with small numbers of studies. Forest plots were generated for visual presentation, and the quality of evidence was assessed using the Grading of Recommendations Assessment, Development, and Evaluation (GRADE) framework [31].

Results

Literature Search Flow and the Retrieved Studies

**Figure 1:**
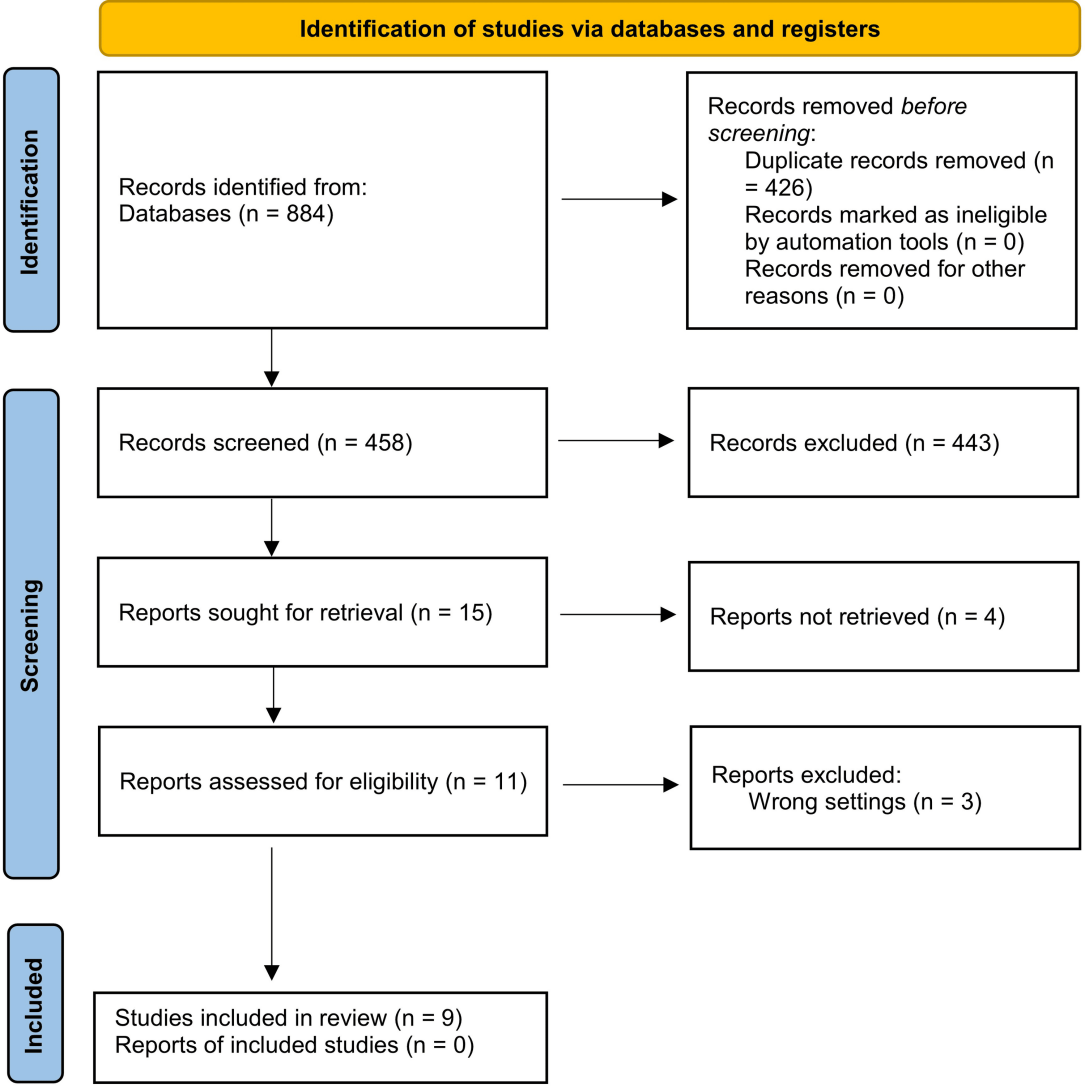
The Preferred Reporting Items for Systematic Reviews and Meta-Analyses (PRISMA) 2020 flow diagram of studies' identification, screening, and inclusion into the review.

An electronic search across various databases and reference lists produced 884 references. After eliminating duplicates, 458 citations were carefully reviewed. Subsequently, 443 documents were excluded after screening their titles and abstracts, leaving 11 full-text records for eligibility assessment. Ultimately, the systematic review included nine studies [14,20,25,27,32-36]. The PRISMA flow diagram illustrating the inclusion and selection process is presented in Figure 1. Excluded articles after full-text assessment, with reasons for exclusion, are illustrated in appendix 2.

Characteristics of Included Studies

A total of nine RCTs were included in this systematic review, all of which investigated various acceleration methods for en masse retraction in orthodontic treatment [14,20,25,27,32-36]. The characteristics of those included trials are illustrated in Table 3.

**Table 3:** Characteristics of included studies in the systematic review. M/F: male/female; Mal: malocclusion; LIES: low-intensity electrical stimulation; CG: control group; PO: primary outcomes; SO: secondary outcomes; RTM: rate of tooth movement; PRP: platelet-rich plasma; CAPs: corticoalveolar perforations; TC: traditional corticotomy; FC: flapless corticotomy; LLLT: low-level laser therapy; AT: appliance type; ERM: en masse retraction method; SS: stainless steel; FA: force application ImageJ software (Bethesda, MD: National Institutes of Health); Viewbox software (Kifissia, Greece: dHAL Software)

Studies	Treatment groups/malocclusion	Appliance characteristics	Participants		Type of interventions	Follow-up time	Outcomes	Methods of primary outcome measurements
			Patient sample/mean age (years)	Gender M/F				
Mousa et al., 2025 [36], Damascus, Syria	Group 1: FC+LLLT, group 2: FC, group 3: CG Mal	AT: MBT 0.022-inch slot size. ERM: sliding technique on 0.019 × 0.025-inch SS archwires with NiTi closed coil springs, FA: 250 g per side	N=63, 21.46±3.16	14/47	FC: vertical incisions (5 mm long, 3 mm deep) on buccal and palatal aspects using a piezosurgery microsaw. LLLT: GaAlAs diode laser (808 nm, 1.1 W, 4 J/point) applied at 32 points (buccal/palatal) every 15 days until retraction completion	Until the canines reach a Class I relationship with normal overjet and overbite, approximately 4 months	PO: RTM, SO: changes in the first molar position, intercanine width, and intermolar width	Digital photographs of plaster models were analysed using ImageJ software
Shaadouh et al., 2024 [20], Damascus, Syria	Group 1: LIES, group 2: CG Mal: Class II division 1	AT: MBT 0.022-inch slot size, ERM: using 0.019 × 0.025-inch SS with NiTi closed coil springs, FA: 250 g per side	N=38, 21.1±2.31	8/30	LIES: electrical stimulation of 15-20 μA was applied to the upper anterior teeth with a removable device before the intervention	Until the canines reach a Class I relationship with normal overjet and overbite, approximately 4 months	PO: RTM, SO: changes in the first molar position, intercanine width, and intermolar width	Digital photographs of plaster models were analyzed using ImageJ software
Al-Bozaie et al., 2024 [25], Aleppo, Syria	Group 1: PRP, group 2: CG Mal: Class II division 1	AT: MBT 0.022 × 0.028-inch slot size, ERM: rigid sectional stainless steel archwire (0.021 × 0.025 inch) for the anterior six teeth with NiTi closed coil springs, FA: 175 g per side	PRP: 21.73±1.79, CG: 22.46±3.24	7/23	Palatal injection of PRP immediately before starting en masse retraction of upper anterior teeth	Until the middle of the en masse retraction, approximately 4 months	PO: RTM, SO: type of tooth movement (controlled tipping versus translation)	Measured clinically using a digital caliper
Kumar et al., 2024 [33], Maharashtra, India	Group 1: CAPs, group 2: CG Mal: Class I bimaxillary protrusion	AT: MBT 0.022-inch slot size, ERM: using 0.019 × 0.025-inch SS with NiTi closed coil springs, FA: 150 g per side	N=30, CAPs: 19.5±2.67, CG: 20.3±2.23	7/13	CAPs were performed at the beginning of space closure and one month after starting the en masse retraction	4 months	PO: RTM, SO: pain perception	Measured on 3D digital models
Chandak and Patil, 2022 [32], Maharashtra, India	Group 1: PRP, group 2: CG Mal: Class I bimaxillary protrusion or crowding	AT: MBT 0.022 × 0.028-inch slot size, ERM: using 0.019 × 0.025-inch SS with NiTi closed coil springs, FA: 150 g per side	N=20, PRP: 20.7±2.35, CG: 20.70±2.31	7/13	PRP was injected submucosally into the anterior maxillary alveolar mucosa at the commencement of retraction	3 months	PO: RTM, SO: anchorage loss, type of anterior retraction, pain assessment	Measured on 3D digital models
Khlef and Hajeer, 2022 [14], Damascus, Syria	Group 1: TC, group 2: FC Mal: Class II division 1	AT: MBT 0.022-inch slot size, ERM: sliding technique on 0.019 × 0.025-inch SS archwires with NiTi closed coil springs, FA: 250 g per side	N=38, TCG: 23.84±3.55, FCG: 23.29±3.60	3/35	TC: vertical soft tissue incisions were made on the buccal and palatal gingiva, followed by piezosurgery incisions in the cortical bone without elevating a flap. FC: full-thickness mucoperiosteal flap elevation was performed, followed by piezosurgery incisions in the cortical bone. Retraction commenced 4 days post-corticotomy	Until the canines reach a Class I relationship with normal overjet and overbite	PO: RTM, amount of en masse retraction, SO: pain perception	Digital photographs of plaster models analyzed using ImageJ software
Raghav et al., 2021 [34], UP, India	Group 1: CAPs, group 2: CG Mal: Class I bimaxillary protrusion or Class II division 1 malocclusion, Irregularity index <4 mm	AT: MBT 0.018-inch slot size, ERM: using 0.016 × 0.022-inch SS with NiTi closed coil springs, FA: 200 g per side	N=60, CAPs: 20.17±2.47, CG: 20.86±2.38	33/27	CAPs: five CAPs were performed per side just before en masse retraction. CAPs were created using a lance pilot drill (2 mm width, 5 mm depth). En masse retraction was initiated immediately after the CAPs	4 months	PO: RTM during en masse anterior retraction, SO: molar anchorage loss	Measured on 3D digital models
Khlef et al., 2020 [35], Damascus, Syria	Group 1: TC, group 2: FC Mal: Class II division 1	AT: MBT 0.022-inch slot size, ERM: sliding technique on 0.019 × 0.025-inch SS archwires with NiTi closed coil springs, FA: 250 g per side	N=40, TCG: 22.44±3.55, FCG: 21.90±3.60	4/36	TC: patients had traditional corticotomy with full-thickness flap elevation and cortical incisions using piezosurgery, FC: patients had flapless corticotomy with vertical soft tissue and cortical incisions by piezosurgery, with no flap elevation. Retraction began 4 days post-corticotomy	Until the canines reach a Class I relationship with normal overjet and overbite (4.04±1.10 months for the FCG and 3.75±2.14 months for the TCG)	PO: duration of en masse retraction. SO: skeletal, dental, and soft-tissue changes assessed, external apical root resorption of maxillary anterior teeth	Digital photographs of plaster models were analyzed using Viewbox software
Hatrom et al., 2020 [27], Jeddah, Saudi Arabia	Group 1: FC, group 2: CG Mal: Class II division 1	AT: modified bidimensional multibracket system with: 0.018-inch slots for incisors and canines, 0.022-inch slots for premolars and molars, ERM: using 0.018 × 0.025-inch SS archwire with NiTi closed-coil springs, FA: 250 g per side	N=26, FC: 19.8±3.1, CG: 20.4±4.1	11/12	FC: piezocision was performed using a piezoelectric knife to create cortical alveolar incisions on the buccal side. Activation time: en masse retraction was initiated one week after extraction and piezocision	4 months	PO: amount of en masse retraction, type of tooth movement, root resorption, pain levels. SO: extraction space closure, rate of space closure	Measured on 3D digital models

The nine included studies enrolled 347 patients (82 males {23.6%} and 265 females {76.4%}), with a mean age of approximately 21.6±2.97 years (range: 13-30 years). Sample sizes varied from 20 to 63 participants, and all trials included both genders, though females predominated in most studies.

Among these, eight studies were two-arm trials [14,20,25,27,32-35], and one was a three-arm trial [36]. Seven studies compared an experimental group to a control group [14,20,25,27,32,34,35], while one study compared two different experimental groups [33]. The remaining study included two experimental and one control group [36]. Six studies included patients with Class II, division 1 malocclusion who required premolar extractions [14,20,25,27,35,36]. Three studies included patients with Class I malocclusion with bimaxillary protrusion [32-34].

The interventions evaluated in these studies included traditional corticotomy (TC) [14,35], flapless corticotomy (FC) [14,27,35,36], corticoalveolar perforations (CAPs) or the so-called micro-osteoperforations [33,34], low-intensity electrical stimulation [20], platelet-rich plasma (PRP) injections [25,32], and one trial combined FC and LLLT [36].

The primary outcome across all studies was the rate of en masse retraction (RER), typically reported in millimeters per month (mm/month). This was measured using various methods, including study models with digital photographs [14,20,36], 3D scanning of plaster models [32-34], digital calipers for space closure distance [25], cephalometric analysis [35], and cone-beam computed tomography (CBCT) [27].

The duration of follow-up in these studies was determined by the achievement of specific clinical endpoints. Among the reviewed studies, seven trials maintained their follow-up until the conclusion of en-masse retraction, specifically when a Class I canine relationship was achieved or when complete closure of extraction spaces was achieved [14,20,35,36]. Three studies reported a standardized follow-up period of four months [27,33,34], while one trial conducted assessments every 40 days until the estimated midpoint of the retraction period was reached [25]. Additionally, one study followed patients for only three months [32].

Risk of Bias of Included Studies

**Figure 2:**
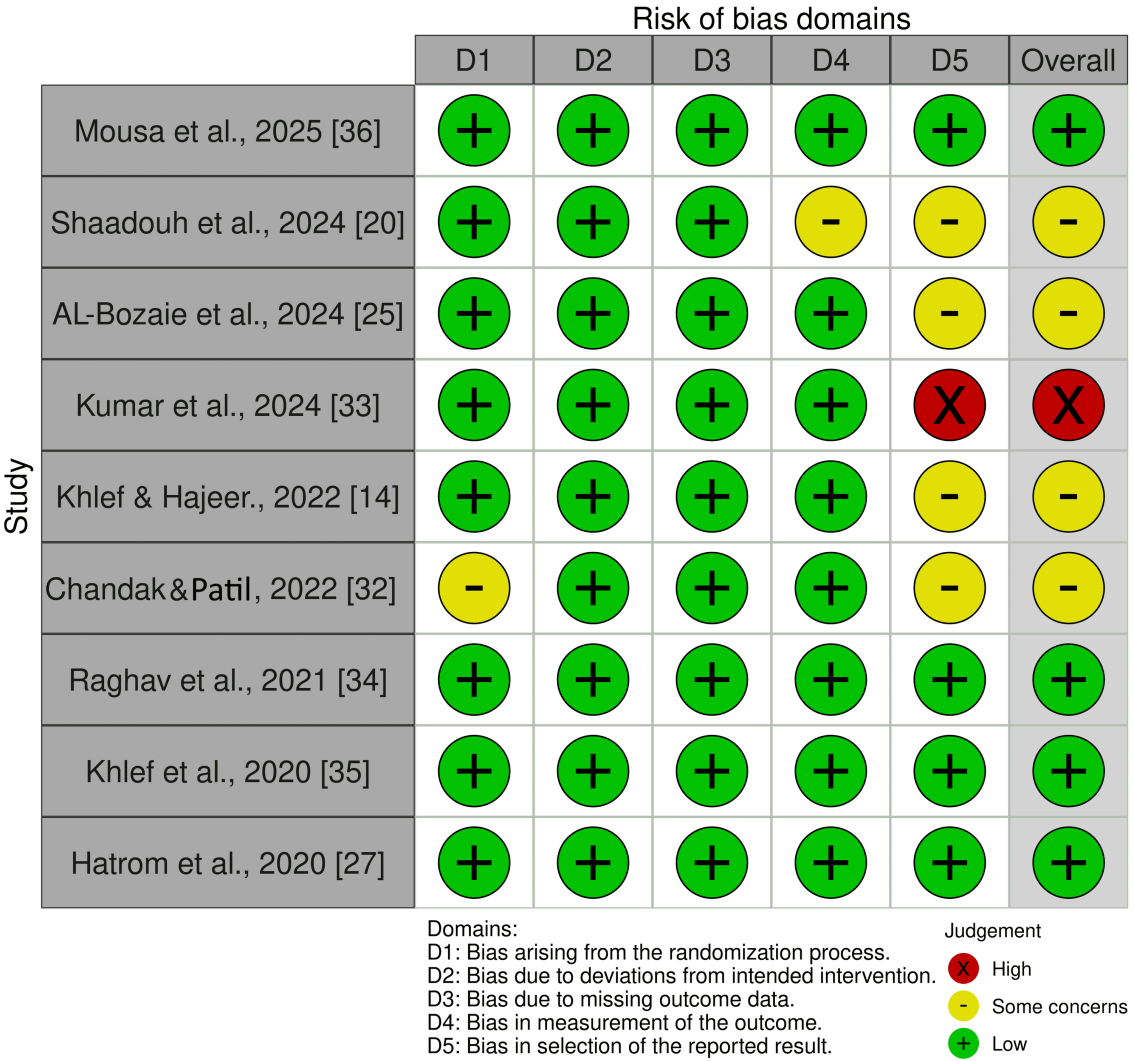
Risk of bias summary: review authors’ judgments for each risk of bias domain for the included studies.

**Figure 3:**
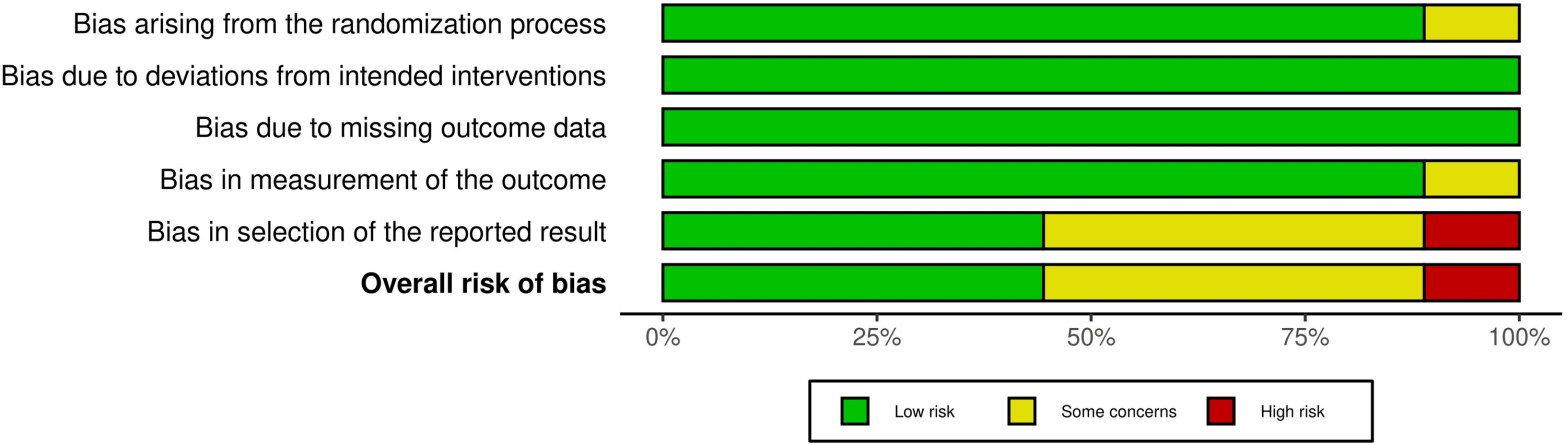
Risk of bias graph: review authors’ judgments about each risk of bias item, presented as percentages across all included studies.

Figures 2, 3 summarize the overall risk of bias for the included studies. One RCT was classified as having a high risk of bias [33]. However, four RCTs were classified as having some concerns of bias [14,20,25,32], while the remaining studies were judged to be at low risk of bias [27,34-36].

The reasons for the high risk of bias in the mentioned RCT include the selection of the reported result due to retrospective registration and the significant increase in sample size, which raises serious concerns about the selection of the reported result [33]. The reasons for some concerns of bias in the four mentioned RCTs include: (1) incomplete descriptions of the randomization process [32]; (2) measurement of the outcome [20]; (3) selection of the reported result [20,25,32,35]. Additional details about assessing the risk of bias are provided in appendix 3.

Effects of Interventions

A synopsis of quantitative measurements in each study is presented in Table 4. The certainty of evidence for each intervention, assessed using the GRADE framework, is summarized in Table 5.

**Table 4:** Summary of main findings. LIES: low-intensity electrical stimulation; CG: control group; RTM: rate of tooth movement; PRP: platelet-rich plasma; CAPs: corticoalveolar perforations; TCG: traditional corticotomy group; FCG: flapless corticotomy group; LLLT: low-level laser therapy; CONV: conventional retraction

Studies	Statistical significance of reported primary outcomes
Mousa et al., 2025 [36] Damascus, Syria	RTM: FC+LLLT group: 1.32±0.19 mm/month (43.8% acceleration versus CONV), FCG: 1.09±0.13 mm/month (31.8% acceleration versus CONV), CG: 0.75±0.06 mm/month. Significance: p<0.001, FC+LLLT>FC>CONV
Shaadouh et al., 2024 [20] Damascus, Syria	RTM: LIES: 1.02±0.08 mm/month, CG: 0.73±0.04 mm/month, significance: p<0.001, statistically significant but may lack clinical relevance
Al-Bozaie et al., 2024 [25] Aleppo, Syria	RTM: PRP: 3.30±1.15 mm, CG: 3.09±0.99 mm, no significant difference (p=0.596)
Kumar et al., 2024 [33] Maharashtra, India	CAPs increased the rate by 1.53-fold in the maxilla and 1.39-fold in the mandible compared to controls. Significant acceleration during both CAPs (T₀-T₂) and post-CAPs (T₂-T₄) periods (p<0.05)
Khlef and Hajeer, 2022 [14] Damascus, Syria	RTM: TCG was greater in the first 3 months (1.82, 1.66, 1.39 mm/month) versus FCG (1.60, 1.42, 1.22 mm/month), with statistically significant differences (p<0.001 for all comparisons).
Chandak and Patil, 2022 [32] Maharashtra, India	PRP: 2.10±0.41 mm, CG: 2.07±0.19 mm, p=0.838 (not significant)
Raghav et al., 2021 [34] UP, India	1st month: CAPs: 0.71±0.19 mm, CG: 0.43±0.20 mm, p=0.001 (significant), months 2-4: no significant differences (p>0.05)
Khlef et al., 2020 [35] Damascus, Syria	Retraction duration: FCG: 4.04±1.10 months, TCG: 3.75±2.14 months, no significant difference: (95% CI: -0.81 to 1.39; p=0.59)
Hatrom et al., 2020 [27] Jeddah, Saudi Arabia	RTM: FCG: 1.2 mm/month versus CG: 0.6 mm/month (p<0.001 for all comparisons)

**Table 5:** Summary of findings according to the GRADE guidelines for the included trials. High quality: further research is very unlikely to change our confidence in the estimate of effect. Moderate quality: further research is likely to have an important impact on our confidence in the estimate of effect and may change the estimate. Low quality: further research is very likely to have an important impact on our confidence in the estimate of effect and is likely to change the estimate. Very low quality: we are very uncertain about the estimate. Decline one level for risk of bias. Decline one level for inconsistency (I^2^=54%). Decline one level for risk of bias {bias in the selection of reported results [33]}, and decline one level for inconsistency (I^2^=50%). Decline two levels for risk of bias {bias in the measurement of the outcomes and selection of reported results [20]}. Decline two levels for risk of bias {incomplete descriptions of the randomization process [32], Selection of the reported result [25]}. CI: confidence interval; PG: parallel-group design; RER: rate of en-masse retraction; FC: flapless corticotomy; MD: mean difference; CONT: control group; LLLT: low-level laser therapy; CAPs: corticoalveolar perforations; LIES: low-intensity electrical stimulation; PRP: platelet-rich plasma; GRADE: Grading of Recommendations Assessment, Development, and Evaluation; RCT: randomized controlled trial

Quality assessment criteria							Summary of findings				Comments
No. of studies		Risk of bias	Inconsistency	Indirectness	Imprecision	Other considerations	No. of patients	Effects		Certainty	
								Absolute (95% CI)	Relative (95% CI)		
RER associated with FC											
FC versus TC	2 RCTs (PG)	Serious	Not serious	Not serious	Not serious		78	-	-	⨁⨁⨁◯^a^ Moderate	The RER was greater in TC group compared to the FC group; this difference was not statistically significant, with a moderate quality of evidence ⨁⨁⨁◯
FC versus CONT	2 RCTs (PG)	Not serious	Serious	Not serious	Not serious		89	-	MD=0.24; 95% CI: 0.15, 0.34;	⨁⨁⨁◯^b^ Moderate	In two studies, flapless corticotomy enhanced the RER with varying acceleration ratios, with a moderate quality of evidence ⨁⨁⨁◯
RER associated with a combination of FC and LLLT											
1 RCT (PG)		Not serious	Not serious	Not serious	Not serious		63	-	-	⨁⨁⨁⨁ High	FC+LLLT increased RER by 43.8% compared to conventional methods (high-quality evidence from a single RCT ⨁⨁⨁⨁); however, confirmation through independent multicenter studies is required
RER associated with CAPs											
2 RCT (PG)		Serious	Serious	Not serious	Not serious		90	-	MD=0.28; 95% CI: 0.19, 0.37	⨁⨁◯◯^c^ Low	Both studies showed a significant increase in RER in the first month of retraction, with a low quality of evidence ⨁⨁◯◯
RER associated with LIES											
1 RCT (PG)		Very serious	Not serious	Not serious	Not serious		38	-	-	⨁⨁◯◯^ d^ low	That LIES significantly increased the RER by approximately 28% compared to traditional methods, with a low quality of evidence ⨁⨁◯◯
RER associated with PRP											
2 RCT (PG)		Very serious	Not serious	Not serious	Not serious		50	-	-	⨁⨁◯◯^e^ Low	Both studies concluded that PRP is ineffective in accelerating en masse retraction, with a low quality of evidence ⨁⨁◯◯

Flapless Versus Traditional Corticotomy

Two RCTs investigated the comparative efficacy of FC and TC in accelerating en masse retraction, with outcomes demonstrating methodological dependency [14,35]. These two studies were excluded from the meta-analysis due to variations in the variables under investigation. Khlef and Hajeer and Khlef et al. directly compared FC and TC protocols. Their findings revealed statistically significant differences in monthly retraction rates during the initial three-month period, with TC demonstrating superior performance (1.82±0.21 mm, 1.66±0.18 mm, and 1.39±0.15 mm) compared to FC (1.60±0.19 mm, 1.42±0.17 mm, and 1.22±0.14 mm; p<0.001) [14]. However, the treatment duration showed no significant intergroup difference (FC: 4.04±1.10 months versus TC: 3.75±2.14 months; p=0.59), indicating comparable long-term efficacy despite early-phase differences; the strength of evidence was moderate [35].

Flapless Corticotomy Versus Non-accelerated Treatment

**Figure 4:**
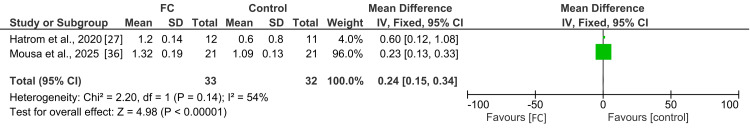
Forest plot of the comparison between flapless corticotomy group and control group for rate of en-masse retraction. FC: flapless corticotomy

Two trials evaluated FC against conventional en masse retraction [27,36]. The pooled estimate showed that the FC increased the RER by 0.24 mm/month (MD=0.24; 95% CI: 0.15-0.34; p<0.0001), with moderate but statistically significant heterogeneity (χ²=2.20, p=0.014; I²=54%), indicating variability in effect sizes between studies (Figure 4). The certainty of evidence was rated as moderate.

Combination of Flapless Corticotomy and Low-Level Laser Therapy Protocol

An RCT conducted by Mousa et al. assessed the combined intervention FC and LLLT, which achieved a notable retraction rate of 1.32±0.19 mm/month. This represented a 43.8% acceleration compared to conventional methods with retraction rates of 0.75±0.06 mm/month (p<0.001) [36]. Moreover, comparative analysis revealed that the FC+LLLT protocol resulted in a retraction rate that was 21.1% higher than that of FC alone, which recorded a rate of 1.09±0.13 mm/month (p<0.001). A temporal analysis demonstrated distinct patterns of efficacy. While the acceleration observed in the FC group was limited to the initial two-month period, the FC+LLLT group maintained its elevated retraction rate throughout the treatment [36]. The strength of the evidence was high.

Given that conventional en masse retraction typically requires six to 10 months, the 43.8% acceleration with FC+LLLT translates to a reduction of approximately 2.6-4.4 months, which substantially exceeds the minimal important difference threshold. This finding, supported by high-quality GRADE evidence from a single RCT, underscores the clinical promise of this combined approach while highlighting the need for confirmation in future multicenter studies.

Corticoalveolar Perforations

**Figure 5:**
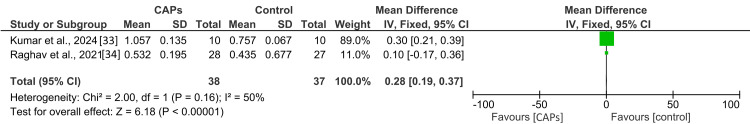
Forest plot of the comparison between corticoalveolar perforations and control group for rate of en masse retraction. CAP: corticoalveolar perforations

Two trials examined the effect of CAPs on the RER, yielding mixed results [33,34]. The pooled analysis indicated that CAPs increased the RER by 0.28 mm/month (MD=0.28; 95% CI: 0.19-0.37; p<0.0001), with moderate but statistically significant heterogeneity (χ²=2.00, p=0.016; I²=50%; Figure 5). The certainty of evidence was rated as low.

Low-Intensity Electrical Stimulation

One trial by Shaadouh et al. examined the effects of LIES on en masse retraction. The authors demonstrated that LIES significantly increased the RER by approximately 28% compared to traditional methods (1.02±0.08 mm/month versus 0.73±0.04 mm/month; p<0.001) [20]. The strength of evidence was low.

Platelet-Rich Plasma Injections

Two studies examined the effects of PRP injection on en masse retraction [25,32]. The meta-analysis was not conducted due to heterogeneity between the two studies regarding the injection site and the biomechanical system. One study employed a segmented mini-implant technique [25], while the other used a continuous technique [32].

Chandak and Patil and Al-Bozaie et al. found that PRP did not significantly enhance RER compared to the control group. Specifically, Chandak and Patil noted a negligible increase in overall RER in the experimental group (2.10±0.41 mm) compared to the control group (2.07±0.19 mm; p=0.838) [32]. Similarly, Al-Bozaie et al. reported an RER of 0.82 mm/month in the PRP group, while the control group demonstrated an RER of 0.77 mm/month (p=0.596) [25]. Both studies concluded that PRP is ineffective in accelerating orthodontic tooth movement during en masse retraction [25,32]; the strength of evidence was low.

Discussion

This review aimed to comparatively evaluate the existing evidence on the effectiveness of different methods for accelerating the en masse retraction of upper anterior teeth. It consolidates data from nine RCTs involving 347 patients and is based on an initial literature search of 884 studies on accelerated retraction of upper anterior teeth. It should also be noted that most of the included trials were conducted within a limited geographic region (primarily the Middle East), which may limit the extrapolation of these findings to other populations with different genetic backgrounds, dietary habits, or healthcare delivery systems.

Flapless Versus Traditional Corticotomy

Regarding FC versus TC for en masse retraction, Khlef and Hajeer and Khlef et al. demonstrated significantly faster initial monthly retraction rates for TC compared to FC, potentially linked to the greater bone and gingival injuries observed in the TC Group [14,35]. However, the overall mean retraction duration was lower in the TC group (4.22 months) compared to the FC group (4.58 months); notably, both durations represent a substantial reduction (≥45%) compared to conventional non-accelerated protocols documented in other studies (range: 6.08-10.35 months) [36-38]. This level of reduction exceeds the 20-40% threshold defining an accelerated orthodontic technique [8]. Despite the numerical difference in mean duration favoring TC, the difference in overall treatment time between TC and FC was not statistically significant. This indicates that while TC offers an early acceleration advantage, FC achieves comparable long-term efficiency, potentially with reduced morbidity. Consequently, FC emerges as a viable minimally invasive alternative to TC, balancing equivalent overall treatment time against TC's short-term kinetic superiority [14].

Flapless Corticotomy Versus Non-accelerated Treatment

A meta-analysis of two RCTs confirms that FC significantly accelerates orthodontic en masse retraction compared to conventional treatment [27,36]. The pooled mean difference in retraction rate was 0.24 mm/month (95% CI: 0.15-0.34; p<0.0001). While the effect direction was consistent and statistically robust, moderate heterogeneity (I²=54%) indicated significant variation in the magnitude and duration of acceleration across studies. Hatrom et al. reported a 100% increase in velocity, whereas Mousa et al. observed only a 31.8% acceleration, confined to the initial two-month period [27,36]. As a result, the overall strength of evidence for this effect is rated as moderate according to GRADE criteria.

This discrepancy is primarily attributed to biomechanical differences in the appliances' design. Hatrom et al. utilized a bidimensional system (0.018-inch anterior/0.022-inch posterior slots with 0.018 × 0.022-inch wires) [27], which reduces friction and optimizes force distribution, thereby enhancing the expression of the RAP induced by FC [39]. Conversely, Mousa et al. employed a conventional 0.022-inch slot system with stiffer 0.019 × 0.025-inch archwires [36], which is associated with greater frictional resistance [40]. This temporal limitation aligns with the physiological transition of RAP bone remodeling from resorption to formation, which occurs within eight to 12 weeks [41]. Orthodontists generally consider a 20-40% reduction in treatment time a key motivator for adopting such acceleration techniques [8]. Importantly, Mousa’s torque-controlled design likely achieved bodily movement, whereas Hatrom’s friction-prone system may have permitted uncontrolled tipping despite stiffer wires.

Although a 0.24 mm/month increase may appear modest, it translates to approximately 2.4 mm of additional space closure over a 10-month retraction period, potentially reducing overall treatment duration by 1.5 to two months compared to conventional protocols. This reduction is likely to be clinically meaningful for both patients and practitioners, as it falls within the range that most orthodontists consider attractive for adopting acceleration techniques [8].

Combination of Flapless Corticotomy and Low-Level Laser Therapy Protocol

The significant acceleration achieved through the combined protocol of FC+LLLT, demonstrated by a 43.8% increase in the RER compared to conventional methods (p<0.001) [36], warrants careful assessment beyond mere quantitative results. As stated by Mheissen et al., most orthodontists view a 20-40% decrease in treatment time as an attractive motivation for adopting methods to accelerate orthodontic tooth movement; notably, one month is the minimal important difference for the reduction in treatment duration [42]. As such, acceleration procedures that may reduce treatment duration to less than one month, even if statistically significant, may not be important for many patients [43].

Given that conventional en masse retraction typically requires six to 10 months, the 43.8% acceleration with FC+LLLT (representing a reduction of ~2.6-4.4 months) far exceeds the minimal important difference threshold and therefore represents a highly compelling outcome, despite a low strength-of-evidence rating based on GRADE criteria.

Unlike the temporary advantages of flapless corticotomy alone, which were limited to the initial two months, the effects of FC+LLLT were maintained at an accelerated rate throughout the entire treatment period. This sustained effect may be explained by the immunomodulatory action of low‑level laser therapy, which reduces pro‑inflammatory cytokines (e.g., IL‑1β, TNF‑α) and promotes anti‑inflammatory mediators [44,45]. By alleviating the acute inflammatory response and fostering an optimal environment for bone remodeling, LLLT effectively extends the RAP initiated by corticotomy [46,47].

Low-Intensity Electrical Stimulation

The findings from the trial conducted by Shaadouh et al. revealed that the en masse retraction rate in the LIES group was statistically significantly greater at all measured time points compared to the control group (p<0.001) [48,49]. This suggests that low-intensity direct electric stimulation is an effective method for promoting orthodontic tooth movement by 28%. However, the strength of evidence for this effect is rated as very low according to GRADE criteria. Although this 28% acceleration was statistically significant, the authors concluded that it may not have substantial clinical implications given the small magnitude of the difference (0.29 mm/month) [20]. Nevertheless, LIES appears to be effective in promoting orthodontic tooth movement. These results are in agreement with Kim et al.’s study, which reported a roughly 30% increase in the rate of canine retraction with the daily application of low-intensity direct electrical stimulation at 15 µA [48]. Additionally, this finding aligns with the earlier pilot study by Shaadouh et al., which reported a total retraction rate of approximately 0.97±0.06 mm/month, closely matching the current study's rate of 1.02±0.08 mm/month [49].

Corticoalveolar Perforations

Meta-analysis pooled estimates indicated that CAPs significantly accelerated orthodontic retraction by a mean difference of 0.28 mm/month (95% CI: 0.19-0.37; p<0.0001) compared to conventional treatment, suggesting clinically meaningful acceleration. However, this conclusion requires cautious interpretation due to moderate statistical heterogeneity (I²=50%) and a critically low strength-of-evidence rating based on GRADE criteria, reflecting methodological limitations in the literature. Discrepancies in outcomes across key studies underscore the efficacy that is dependent on specific protocols. Raghav et al. noted a transient acceleration limited to the initial month, with no significant differences observed in subsequent months (p>0.05) [34]. In contrast, Kumar et al. reported sustained acceleration over four months when comparing maxillary and mandibular RER with a control group [33]. These differing outcomes may be attributed to variations in protocol and biological mechanisms. Raghav et al.'s method involved a single application of CAPs (with five perforations per side), corresponding to the acute inflammatory phase of the RAP, during which cytokine-mediated osteoclast activation drives the initial acceleration [34,50]. However, the transient nature of their results suggests that the RAP effects were not sustainable. Conversely, Kumar et al.'s approach involved 21 repeated CAPs, implemented at the beginning of space closure and at the first monthly follow-up, as well as extended perforation sites (interdental and distal to all anterior teeth) [33], likely prolonging the effects of RAP through sustained cytokine release. This is consistent with evidence indicating that the frequency and density of CAP applications are linked to remodeling duration [51]. Moreover, Kumar et al.'s perforations (4 mm depth) were deeper than those of Raghav et al. (5 mm depth but only 2 mm wide) [33,34], potentially facilitating better access to cancellous bone, enhancing osteoclastic recruitment [52]. Notably, Kumar et al. observed greater acceleration in the maxilla compared to the mandible, likely reflecting the lower density and faster remodeling rates of maxillary bone [33].

Platelet-Rich Plasma Injections

The consistent findings from two randomized trials demonstrate that PRP injections do not significantly accelerate the rate of RER [25,32]. Despite methodological heterogeneity, including divergent injection sites (palatal versus buccal mucosa) and biomechanical systems (segmented arch/mini-implant versus continuous arch), both studies reported nearly identical RERs between the PRP and control groups [25,32]. This convergence of null outcomes across distinct protocols suggests that PRP’s inefficacy is independent of technical variables. However, the strength of evidence for this effect is rated as low according to the GRADE criteria.

Biologically, PRP's ineffectiveness in enhancing tooth movement contradicts its theoretical mechanism. PRP is believed to stimulate osteoclastogenesis through growth factors such as PDGF and TGF-β, as well as cytokines, thereby triggering the RAP [32]. However, the observed outcomes are consistent with evidence suggesting that PRP may activate compensatory feedback mechanisms. Elevated growth factor concentrations can inhibit the production of endogenous cytokines [53], while systemic changes in inflammatory markers (e.g., alkaline phosphatase, gamma-glutamyl transferase) may hinder local bone remodeling [54].

Limitations

Despite including nine RCTs, this systematic review has several limitations. First, the heterogeneity in intervention protocols, biomechanical designs, and measurement techniques limited the ability to perform a quantitative synthesis of the collected data. Second, some studies were rated as having high risk or some concerns of bias stemming from incomplete randomization details, retrospective registration, and selective outcome reporting, which may compromise the reliability of their findings. Third, most included trials were conducted in the Middle East, which limits the generalizability of our findings to other populations. Future multicenter studies with broader geographic representation are needed to confirm these results. Finally, variability in outcome assessment methods (including digital models, cephalometric tracings, and CBCT), patient demographics (age ranges and sex distribution), and anchorage systems introduces additional confounders that may limit the generalizability of the results.

## Conclusions

This review identified several surgical and non-surgical techniques that demonstrate a statistically significant acceleration in orthodontic tooth movement. Flapless corticotomy, low-intensity electrical stimulation, and corticoalveolar perforations all showed higher retraction rates than conventional treatment. The most substantial acceleration was observed when flapless corticotomy was combined with low-level laser therapy. However, the clinical interpretation of these findings requires caution. The overall quality of evidence was low to moderate for most techniques, and small sample sizes, methodological heterogeneity, and the fact that a significant proportion of studies on flapless corticotomy were conducted by the same research team introduce potential bias and limit the generalizability of the results. Therefore, while promising, the current evidence does not allow for a definitive conclusion regarding the clear superiority of one technique over another. Any perceived clinical benefits must be weighed against the invasiveness, cost, and potential risks of each procedure.

The limitations of the current evidence base underscore the need for more robust and methodologically sound investigations. Future high-quality, randomized controlled trials with larger sample sizes and longer follow-up periods are essential. There is a critical need for independent, multicenter studies to validate findings, particularly for flapless corticotomy. Research should implement standardized acceleration protocols, consistent biomechanical setups, and uniform outcome measures to facilitate more meaningful comparisons and powerful meta-analyses. A particular priority is for direct comparative studies that contrast multiple acceleration modalities under identical clinical conditions. Furthermore, incorporating patient-centered endpoints - such as levels of discomfort, oral health-related quality of life, and overall treatment satisfaction - will provide a more comprehensive assessment of the true clinical utility and value of these techniques.

## Appendices

Appendix 1 

**Table 6:** Electronic search strategy. RAP: regional acceleratory phenomenon; PAOO: periodontally accelerated osteogenic orthodontics; LLLT: low-level laser therapy; HELT: high-energy laser therapy; YAG: yttrium-aluminum garnets; YSGG: yttrium scandium gallium garnet

Database	Search strategy
CENTRAL (The Cochrane Library)	#1 orthodontic* OR "Tooth movement" OR "orthodontic tooth movement" OR "orthodontic Treatment” OR "orthodontic Therapy". #2 En-mass* OR “extraction therapy” OR “front retraction” OR “orthodontic gap closure” OR “orthodontic space closure”. #3 accelerate* OR rapid* OR short* OR speed* OR fast OR velocity OR duration OR rate OR time OR "regional accelerated phenomenon" OR RAP. 4# corticotom* OR Corticotomy* OR decorticat* OR alveolar Surg* OR PAOO* OR periodontally accelerated orthodontic osteogenesis*. #5 minimally invasive* OR piezosurgery OR piezoelectric OR piezo* OR lasercision OR corticopuncture OR piezocision OR piezotome OR piezopuncture #6 microsurgery OR micro incisions OR micro osteoperforations OR micro perforations OR perforations OR corticision. #7 laser OR “high intensity laser therapy” OR “HELT” OR “hard laser” OR “high-energy laser” OR “Erbium lasers” OR “Er: YAG laser” OR “Er, Cr:YSGG laser” OR “Nd:YAG laser” OR “laser-assisted corticotomy” OR “laser induced” OR “laser decortication” OR “low-level LASER therapy” OR “low-intensity LASER” OR “LLLT”. #8 #4 OR #5 OR #6 OR #7 #9 #1 AND #2 AND #3 AND
Embase	#1 orthodontic* OR "Tooth movement" OR "orthodontic tooth movement" OR "orthodontic Treatment” OR "orthodontic Therapy". #2 En-mass* OR “extraction therapy” OR “front retraction” OR “orthodontic gap closure” OR “orthodontic space closure”. #3 accelerate* OR rapid* OR short* OR speed* OR fast OR velocity OR duration OR rate OR time OR "regional accelerated phenomenon" OR RAP. 4# corticotom* OR Corticotomy* OR decorticat* OR alveolar Surg* OR PAOO* OR periodontally accelerated orthodontic osteogenesis*. #5 minimally invasive* OR piezosurgery OR piezoelectric OR piezo* OR lasercision OR corticopuncture OR piezocision OR piezotome OR piezopuncture #6 microsurgery OR micro incisions OR micro osteoperforations OR micro perforations OR perforations OR corticision. #7 laser OR “high intensity laser therapy” OR “HELT” OR “hard laser” OR “high-energy laser” OR “Erbium lasers” OR “Er: YAG laser” OR “Er,Cr:YSGG laser” OR “Nd:YAG laser” OR “laser-assisted corticotomy” OR “laser induced” OR “laser decortication” OR “low-level LASER therapy” OR “low-intensity LASER” OR “LLLT”. #8 #4 OR #5 OR #6 OR #7 #9 #1 AND #2 AND #3 AND
PubMed	#1 orthodontic* OR "Tooth movement" OR "orthodontic tooth movement" OR "orthodontic Treatment” OR "orthodontic Therapy". #2 En-mass* OR “extraction therapy” OR “front retraction” OR “orthodontic gap closure” OR “orthodontic space closure”. #3 accelerate* OR rapid* OR short* OR speed* OR fast OR velocity OR duration OR rate OR time OR "regional accelerated phenomenon" OR RAP. 4# corticotom* OR Corticotomy* OR decorticat* OR alveolar Surg* OR PAOO* OR periodontally accelerated orthodontic osteogenesis*. #5 minimally invasive* OR piezosurgery OR piezoelectric OR piezo* OR lasercision OR corticopuncture OR piezocision OR piezotome OR piezopuncture #6 microsurgery OR micro incisions OR micro osteoperforations OR micro perforations OR perforations OR corticision. #7 laser OR “high intensity laser therapy” OR “HELT” OR “hard laser” OR “high-energy laser” OR “Erbium lasers” OR “Er: YAG laser” OR “Er,Cr:YSGG laser” OR “Nd:YAG laser” OR “laser-assisted corticotomy” OR “laser induced” OR “laser decortication” OR “low-level LASER therapy” OR “low-intensity LASER” OR “LLLT”. #8 #4 OR #5 OR #6 OR #7 #9 #1 AND #2 AND #3 AND #8
Scopus	#1 TITLE-ABS-KEY (orthodontic* OR "Tooth movement" OR "orthodontic tooth movement" OR "orthodontic Treatment” OR "orthodontic Therapy"). #2 TITLE-ABS-KEY (En-mass* OR “extraction therapy” OR “front retraction” OR “orthodontic gap closure” OR “orthodontic space closure”). #3 TITLE-ABS-KEY (accelerate* OR rapid* OR short* OR speed* OR fast OR velocity OR duration OR rate OR time OR "regional accelerated phenomenon" OR RAP). #4 TITLE-ABS-KEY (corticotom* OR Corticotomy* OR decorticat* OR alveolar Surg* OR PAOO* OR periodontally accelerated orthodontic osteogenesis*). #5 TITLE-ABS-KEY (minimally invasive* OR piezosurgery OR piezoelectric OR piezo* OR lasercision OR corticopuncture OR piezocision OR piezotome OR piezopuncture*). 6# TITLE-ABS-KEY (microsurgery OR micro incisions OR micro osteoperforations OR micro perforations OR perforations OR corticision). 7# TITLE-ABS-KEY (laser OR “high intensity laser therapy” OR “HELT” OR “hard laser” OR “high-energy laser” OR “Erbium lasers” OR “Er: YAG laser” OR “Er,Cr:YSGG laser” OR “Nd:YAG laser” OR “laser-assisted corticotomy” OR “laser induced” OR “laser decortication” OR “low-level LASER therapy” OR “low-intensity LASER” OR “LLLT”). #8 #4 OR #5 OR #6 OR #7 #9 #1 AND #2 AND #3
Web of Science	#1TS=(orthodontic* OR "Tooth movement" OR "orthodontic tooth movement" OR "orthodontic Treatment” OR "orthodontic Therapy"). #2TS=(En-mass* OR “extraction therapy” OR “front retraction” OR “orthodontic gap closure” OR “orthodontic space closure”). #3TS=(accelerate* OR rapid* OR short* OR speed* OR fast OR velocity OR duration OR rate OR time OR "regional accelerated phenomenon" OR RAP). #4TS=(corticotom* OR Corticotomy* OR decorticat* OR alveolar Surg* OR PAOO* OR periodontally accelerated orthodontic osteogenesis*). #5TS=(minimally invasive* OR piezosurgery OR piezoelectric OR piezo* OR lasercision OR corticopuncture OR piezocision OR piezotome OR piezopuncture*). #6TS=(microsurgery OR micro incisions OR micro osteoperforations OR micro perforations OR perforations OR corticision). #7TS=(laser OR “high intensity laser therapy” OR “HELT” OR “hard laser” OR “high-energy laser” OR “Erbium lasers” OR “Er: YAG laser” OR “Er,Cr:YSGG laser” OR “Nd:YAG laser” OR “laser-assisted corticotomy” OR “laser induced” OR “laser decortication” OR “low-level LASER therapy” OR “low-intensity LASER” OR “LLLT”). #7 #4 OR #5 OR #6 #8 #1 AND #2 AND #3 AND
Google Scholar	#1 (orthodontic OR "Tooth movement") AND (En-mass* OR “extraction therapy” OR “front retraction” OR “orthodontic gap closure” OR “orthodontic space closure”) AND (accelerate OR acceleration OR accelerating OR accelerated OR rapid OR corticotomy OR decortication OR piezosurgery) #2 (orthodontic OR "Tooth movement") AND (En-mass* OR “extraction therapy” OR “front retraction” OR “orthodontic gap closure” OR “orthodontic space closure”) AND (minimally invasive corticotomy OR minimally invasive decortication OR minimally invasive surgery OR minimally invasive piezosurgery ) #3 (orthodontic OR "Tooth movement") AND (En-mass* OR “extraction therapy” OR “front retraction” OR “orthodontic gap closure” OR “orthodontic space closure”) AND (laser OR “high intensity laser therapy” OR “HELT” OR “hard laser” OR “high-energy laser” OR “Erbium lasers” OR “Er: YAG laser” OR “Er,Cr:YSGG laser” OR “Nd:YAG laser” OR “laser-assisted corticotomy” OR “laser induced” OR “laser decortication” OR “low-level LASER therapy” OR “low-intensity LASER” OR “LLLT”). #4

Appendix 2

**Table 7:** Excluded studies and the reasons for exclusion

Study	Reason for exclusion
Kumar et al.: Comparative assessment of the rate of orthodontic tooth movement in adolescent patients undergoing treatment by first bicuspid extraction and en mass retraction, associated with low-frequency mechanical vibrations in passive self-ligating and conventional brackets: a randomized controlled trial. Int Orthod. 2020 Dec;18(4):696-705. doi: 10.1016/j.ortho.2020.08.003. Epub 2020 Nov 6. PMID: 33162347.	Patients under 16 years of age were included.
Lalnunpuii et al.: Comparison of rate of orthodontic tooth movement in adolescent patients undergoing treatment by first bicuspid extraction and en-mass retraction, associated with low level laser therapy in passive self-ligating and conventional brackets: a randomized controlled trial. Int Orthod. 2020 Sep;18(3):412-423. doi: 10.1016/j.ortho.2020.05.008. Epub 2020 Jun 19. PMID: 32571649.	Patients under 16 years of age were included.
Attri et al.: Comparison of rate of tooth movement and pain perception during accelerated tooth movement associated with conventional fixed appliances with micro-osteoperforations - a randomised controlled trial. J Orthod. 2018 Dec;45(4):225-233. doi: 10.1080/14653125.2018.1528746. Epub 2018 Oct 3. PMID: 30281397.	Patients under 16 years of age were included.

Appendix 3 

**Table 8:** Risk of bias of the included randomized controlled trials.

Studies	Randomization process	Deviations from intended interventions	Missing outcome data	Measurement of the outcome	Selection of the reported result	Overall bias
Mousa et al., 2025 [36]	Low risk: the randomization process appears to have been appropriately conducted, and allocation concealment was maintained using sealed envelopes. There is no indication of bias in the randomization process.	Low risk: the blinding of participants and people delivering the intervention cannot be performed. We judge that the outcome is not likely to be influenced by a lack of blinding.	Low risk: no dropouts were reported.	Low risk: the measurement methods were objective and standardized, and the high reliability of the measurements suggests minimal risk of bias.	Low risk: the trial was registered, and the reported outcomes align with the protocol, reducing the risk of selective reporting.	Low risk
Shaadouh et al., 2024 [20]	Low risk: the randomization process appears to have been appropriately conducted, and allocation concealment was maintained using sealed envelopes. There is no indication of bias in the randomization process.	Low risk: the blinding of participants and people delivering the intervention cannot be performed. We judge that the outcome is not likely to be influenced by a lack of blinding.	Low risk: no dropouts were reported.	Some concerns: although the measurement methods were standardized and reliable, the lack of blinding of the outcome assessor could introduce bias in measuring outcomes.	Some concerns: although the trial protocol was registered retrospectively, the outcomes reported in the paper align with the methods described. However, retrospective registration introduces some concerns about selective reporting.	Some concerns
Al-Bozaie et al., 2024 [25]	Low risk: the randomization process appears to have been appropriately conducted, and allocation concealment was maintained using sealed envelopes. There is no indication of bias in the randomization process.	Low risk: the blinding of participants and people delivering the intervention cannot be performed. We judge that the outcome is not likely to be influenced by a lack of blinding.	Low risk: no dropouts were reported.	Low risk: the measurement methods were objective and standardized, and the high reliability of the measurements suggests minimal risk of bias.	Some concerns: although the trial protocol was registered retrospectively, the outcomes reported in the paper align with the methods described. However, retrospective registration introduces some concerns about selective reporting.	Some concerns
Kumar et al., 2024 [33]	Low risk: the randomization process was clearly described, and allocation concealment was maintained using sealed envelopes. There is no indication of bias in the randomization process.	Low risk: the blinding of participants and people delivering the intervention cannot be performed. We judge that the outcome is not likely to be influenced by a lack of blinding.	Low risk: no dropouts were reported.	Low risk: the measurement methods were objective and standardized, and the high reliability of the measurements suggests minimal risk of bias.	High risk: the significant decrease in sample size raises serious concerns about selecting the reported result.	High risk
Khlef and Hajeer, 2022 [14]	Low risk: the randomization process appears to have been properly conducted, and allocation concealment was maintained, reducing the risk of selection bias.	Low risk: the blinding of participants and people delivering the intervention cannot be performed. We judge that the outcome is not likely to be influenced by a lack of blinding.	Low risk: the amount of missing data is small and unlikely to bias the results. The reasons for missing data do not appear to be related to the outcome.	Low risk: the measurement methods were objective and standardized, and the high reliability of the measurements suggests minimal risk of bias.	Some concerns: although the trial protocol was registered retrospectively, the outcomes reported in the paper align with the methods described. However, retrospective registration introduces some concerns about selective reporting.	Some concerns
Chandak and Patil, 2022 [32]	Some concerns: using a lottery method for randomization and the lack of explicit information on allocation concealment raise concerns about the randomization process.	Low risk: the blinding of participants and people delivering the intervention cannot be performed. We judge that the outcome is not likely to be influenced by a lack of blinding.	Low risk: no dropouts were reported.	Low risk: the measurement methods were objective and standardized, and the high reliability of the measurements suggests minimal risk of bias.	Some concerns: information about the registration protocol was mentioned.	Some concerns
Raghav et al., 2021 [34]	Low risk: the randomization process was clearly described, and the use of a lottery method suggests that the allocation was random and unbiased.	Low risk: the blinding of participants and people delivering the intervention cannot be performed. We judge that the outcome is not likely to be influenced by a lack of blinding.	Low risk: no dropouts were reported.	Low risk: the measurement methods were objective and standardized, and the high reliability of the measurements suggests minimal risk of bias.	Low risk: the trial was registered, and the reported outcomes align with the protocol, reducing the risk of selective reporting.	Low risk
Khlef et al., 2020 [35]	Low risk: the randomization process appears to have been properly conducted, and allocation concealment was maintained, reducing the risk of selection bias.	Low risk: the trial was conducted as per the protocol, and there is no evidence of deviations from the intended interventions.	Low risk: no dropouts were reported.	Low risk: the outcomes were measured using objective and standardized methods, and the assessor was blinded, reducing the risk of measurement bias.	Low risk: the trial was registered, and the reported outcomes align with the protocol, reducing the risk of selective reporting.	Low risk
Hatrom et al., 2020 [27]	Low risk: the randomization method (opaque sealed envelopes) is appropriate, and there is no evidence of bias in the randomization process.	Low risk: the trial was conducted as per the protocol, and there is no evidence of deviations from the intended interventions.	Low risk: the missing data were minimal and unlikely to have influenced the results. The study maintained a high level of participant retention.	Low risk: the outcomes were measured using objective and standardized methods, and the assessor was blinded, reducing the risk of measurement bias.	Low risk: the trial was registered prospectively, and the reported outcomes align with the protocol, reducing the risk of selective reporting.	Low risk
